# Supplementary material for: An immersive multisensory virtual reality approach to the study of human-built environment interactions: Technical workflows
Source: MethodsX. 2023 Jul 12;11:102279. doi: 10.1016/j.mex.2023.102279 (PMC10371809; doi:10.1016/j.mex.2023.102279)
Supplement: Supplementary file 1 [file mmc1.docx]

**Article information**

**Article title**

An immersive multisensory virtual reality approach to the study of human-built environment interactions: Technical Workflows

**Authors**

Kun Lyu*

Anastasia Globa

Arianna Brambilla

Richard de Dear

**Affiliations**

School of Architecture, Design and Planning, the University of Sydney

School of Architecture, Design and Planning, the University of Sydney

School of Architecture, Design and Planning, the University of Sydney

School of Architecture, Design and Planning, the University of Sydney

**Corresponding author’s email address and Twitter handle**

[*kun.lyu@sydney.edu.au*](mailto:richard.dedear@sydney.edu.au)

**Keywords**

Virtual Reality, Thermal Sense, Immersive Virtual Environment, Human-computer Interaction

**Related research article**

An immersive multisensory virtual reality approach to the study of human-built environment interactions

[**https://doi.org/10.1016/j.autcon.2023.104836**](https://doi.org/10.1016/j.autcon.2023.104836)

**Abstract**

Virtual Reality technology has gained increased attention due to its capacity to provide immersive and interactive experiences to its users. Although increasing evidence has suggested that incorporating multisensory components in VR can promote the sense of presence and improve user performance, most of the current VR applications are limited to visual and auditory senses. In this article, a novel method of integrating thermal-related devices (heat lamps and fans) into Virtual Reality was developed. Automated interaction with the thermal-related devices was achieved using Arduino-based control module with its program embedded into the VR platform-Unity. The functions, hardware and software requirements of the multisensory Virtual Reality system as well as the step-by-step procedures are detailed to provide a reproducible workflow for future applications.

• A practical workflow to integrate thermal apparatus into Virtual Reality.

• Dynamic airflow and radiative heating incorporated into Virtual Reality.

• Automated process to allow user interaction with the thermal components in Virtual Reality.

**Graphical abstract**


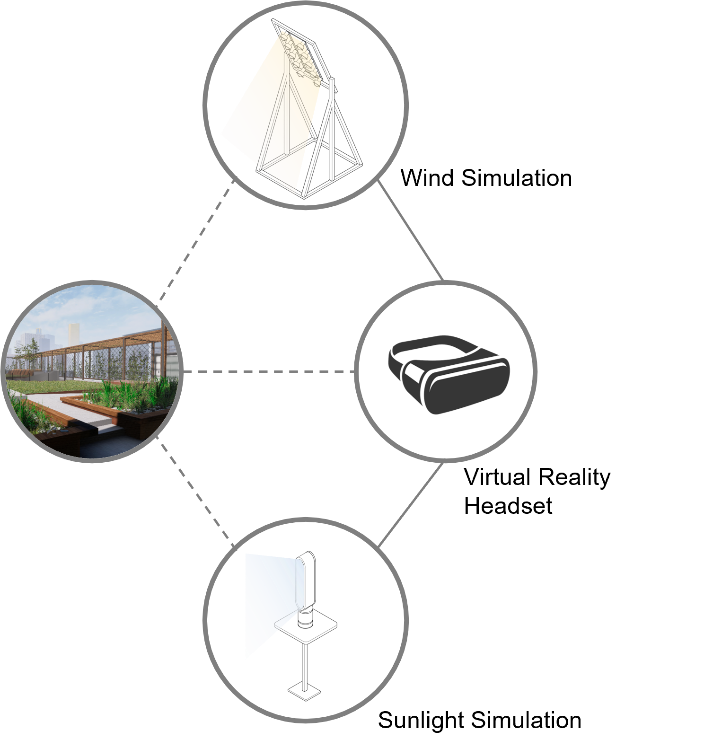


**Specifications table**

| **Subject area** | Engineering |
| --- | --- |
| **More specific subject area** | *Built Environment* |
| **Name of your method** | *Method of Integrating thermal experience in VR* |
| **Name and reference of original method** | *N.A.* |
| **Resource availability** | *Hardware details described in the main article. Software scripts can be found in the supplementary materials.* |

**Method details**

# Introduction

Virtual Reality (VR) technology offers great potential for applications in the field of Architecture, Engineering and Construction as a research method to study human-building interaction (Ergan et al., 2019; Kuliga et al., 2015), as a pre-occupancy evaluation tool (Tseng & Giau, 2022) or as a client engagement tool (Brengman et al., 2022). However, most of the current VR applications have limited capacity, simulating only the visual and auditory environment. Integrating multisensory components (e.g., smell, hot/cold, wind, radiation) into Virtual Reality can improve the sense of presence, *the feeling of being actually there in the virtual environment* (Sanchez-Vives & Slater, 2005) and improve user performance (Cooper et al., 2018). Adding thermal sense to Virtual Reality can open new opportunities. For example, research questions involving human thermal comfort and thermal adaptive behavior in built environments can be studied with this method for the capacity to conduct laboratory-controlled experiment with enhanced sense of immersion and realism to maximize the ecological validity (Alamirah et al., 2022; Smith, 2015). The conventional way of presenting thermal comfort simulation results to stakeholders, which usually relies on graphics and abstract numbers, can be radically transformed by integrating the ability to experientially simulate the embedded multisensory qualities of the design proposal. Various recent studies have explored the possibilities of integrating thermo-haptic feedback into VR, for example, a liquid metal based multimodal sensor and haptic feedback device for thermal and tactile sensation generation in Virtual Reality (Oh et al., 2021), a stretchable copper nanowire-based heater for replication of the feeling of heat in a virtual world (Kim et al., 2020). Besides the thermal hot feeling, recent progresses in VR thermo-haptic demonstrated cold feeling in VR using stretchable skin-like thermoelectric devices (Lee et al., 2020, 2021; Pyun et al., 2022).

This project aims to provide a practical and reproducible workflow for integrating thermal sense into Virtual Reality. The potential applications of the Multisensory Immersive Virtual Environment (MIVE) has been illustrated in (Lyu et al., 2023). The multisensory immersive virtual reality system incorporates the simulation of dynamic airflow and solar radiation into VR and enables the user to effortlessly interact with the thermal environment during their VR experience.

# System Function and Composition

This section describes the function, working mechanism, hardware, and software requirements of the multisensory immersive VR system.

**2.1 Function:**

The multisensory immersive VR system enables the user to perceive a dynamic simulation of sunlight radiation and natural wind from a range of sensory output devices including fans and halogen heat lamps. Moreover, the operation of these sensory output devices can be modified by the VR users, either by navigating to different microclimatic areas in the virtual environment or by modifications of the virtual thermal environment directly in the VR.

**2.2 Working mechanism:**

The multisensory VR system is composed of sensory output devices (VR headset, halogen heat lamps and fans), control modules made from Arduino components (Arduino, 2022a) and integrated development environment of *Unity3D* (2022), see Figure 1.

The intensities of thermal stimuli can be simulated by digitally control the operation of the halogen heat lamps and fans. The frame that mounts the heat lamps is connected to its stand through adjustable hinge joints, allowing users to modify the altitude (elevation) angle of the radiant heat source. This adjustment facilitates the simulation of changing solar angles throughout the year. Users have the freedom to change the relative azimuth angle of the heat lamps by physically orienting themselves in different directions. By defining the relative orientation of the VR avatar to the VR scene and rendering appropriate lighting, it becomes possible to simulate different sunlight angles within a day. Currently, the system utilizes only two fans set in the same direction to simulate wind^[[1]](#footnote-2)^. However, additional fans can be deployed in different directions to provide a more comprehensive simulation of wind from various directions.

Cable protector mats are used to tape-attaching all the exposed cables to the floor in case of possible trapping hazard. Moreover, a playable area (virtual boundary) is defined when starting the VR experience to prevent users from hitting any devices.


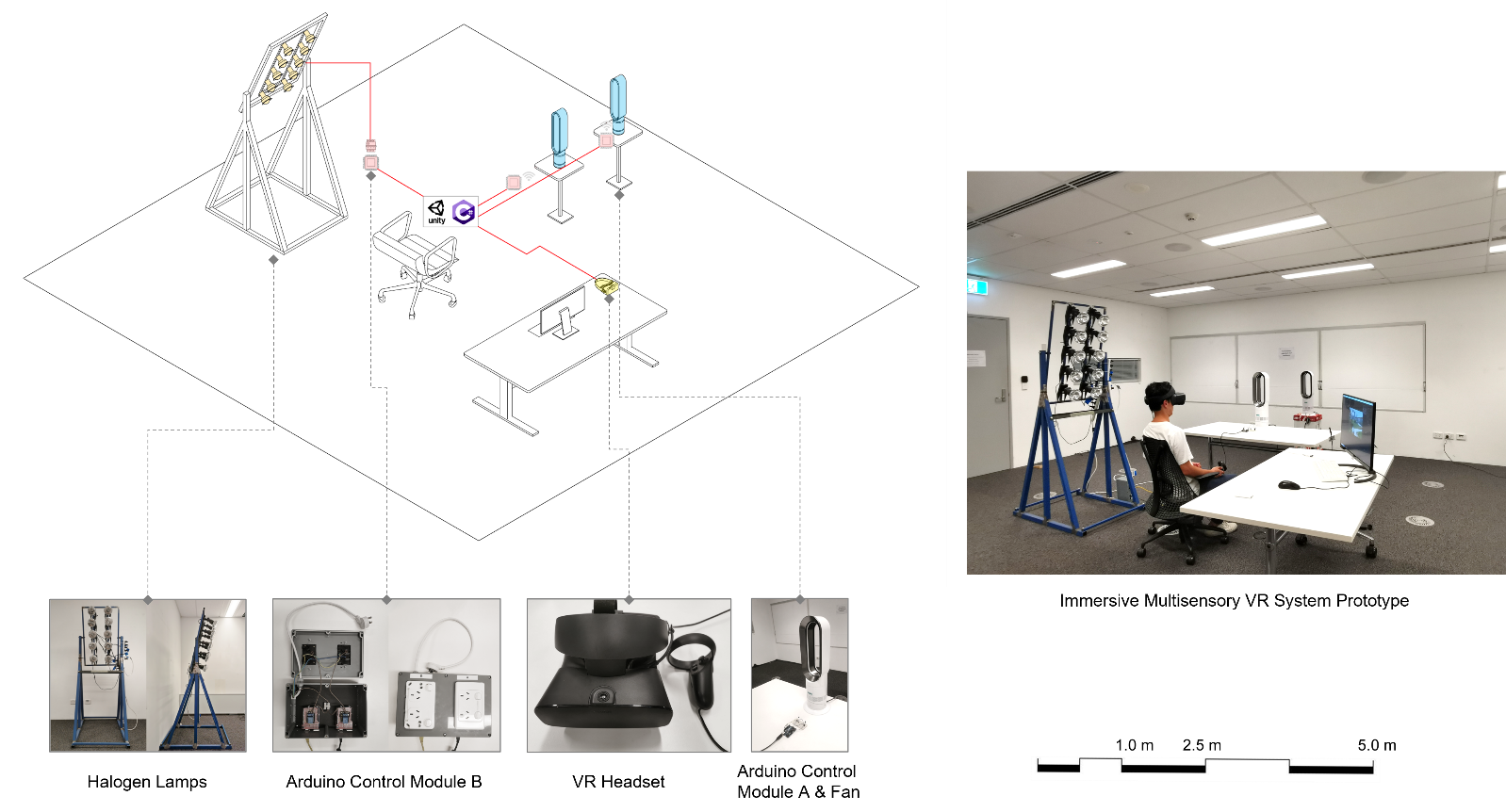


**Figure 1. Multisensory Immersive VR System Composition**

During the experience in VR, thermal-related actions initiated by the users (e.g., moving to shade from sunlight) trigger virtual event function in Unity’s runtime system based on C# language. Signals are sent to the pre-programed control modules to activate certain functions for the operation of the fan or heat lamps (Figure *2*).


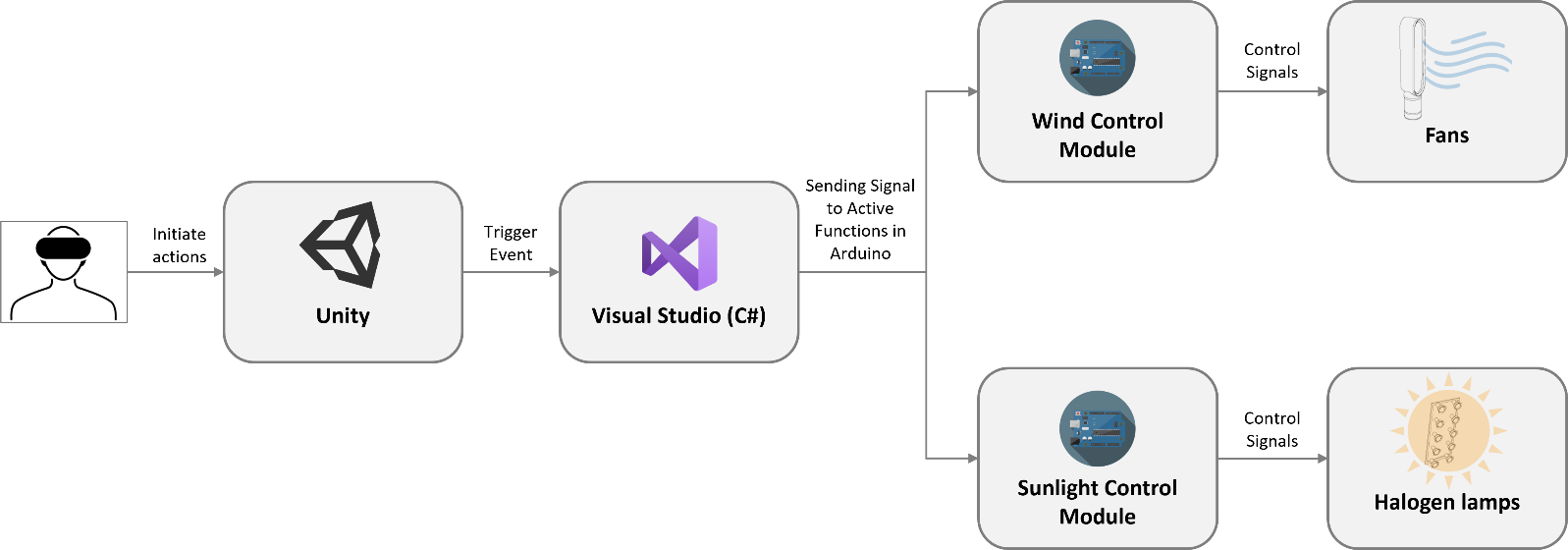


**Figure 2. Working Principle of the Multisensory Immersive VR System**

# Workflow

The process of setting up multisensory VR system can be divided into three steps: *hardware set-up*, *programming Arduino*, *Unity Integration*. This section describes the practical workflow for establishing the thermally integrated VR system. The process of determining the simulated thermal condition is explained in Kun Lyu et al.(2023).

**3.1 Dynamic airflow simulation in VR**

The aim of the dynamic airflow simulation is to: 1) simulate the turbulence characteristics of a time-series recording of actual wind. 2) allow users to have the ability to interact with different airflow profiles during VR experience, e.g., choose different wind profiles or modify wind speed. The fluctuation of air velocity is achieved by transforming the air velocity values into a sequence of control signals for the fans to operate at the respective levels.

***Hardware and Software Components:***

**Table 1. List of Items Required for Wind Simulation in VR**

| **Hardware** | | |
| --- | --- | --- |
| Item | Model | Quantity |
| Virtual Reality headset | Oculus Rift S | 1 |
| VR compatible computer | Lenovo | 1 |
| Bladeless Fan with Remote Control | Dyson AM09 Hot+Cool Fan | 2 |
| Arduino microcontroller | Arduino Uno Rev3 | 2 |
| Solderless Breadboard |  | 2 |
| IR LED Receiver | Adafruit ZD1953 | 2 |
| 5mm Infrared Transmitting LED | Viewing Angle: 30°  Peak Spectral Wavelength (IR): 40nm @ 20mA | 2 |
| Resistor | 10Ω | 2 |
| Jumper wire |  |  |
| Electrical System | Alternating Current system (220-240 Volts, 50Hz) |  |
| **Software** | | |
| Unity 2022 |  |  |
| Visual Studio 2019 |  |  |
| Arduino IDE 2.0 |  |  |

***Step one: Hardware set-up***

The hardware set-up of the dynamic airflow simulation consists of the assembly of the Wind Control Module, fans, and VR headset. The Wind Control Module is made of Arduino UNO Rev3 (microcontroller), solderless breadboard (circuit prototyping board), infrared LED receiver, infrared transmitting LED and resistor. It works as a digitally programed remote control for the operation of the fan to simulate the turbulence characteristics of natural wind. Two functions are achieved, the ability to receive IR signal from the fan’s original remote controller to configure the Arduino Uno Rev3, and the ability to send IR signal to control the fan. Firstly, a series circuit was designed to connect the infrared LED receiver, infrared transmitting LED and the Arduino Rev 3 microcontroller with the Arduino jumper wires (figure 3). Wind Control Module can be connected to the PC from its USB connector so that it can get powered (5v) and receive signal from the serial port in the PC. The infrared transmitting LED has a limited viewing angle (normally 30° - 40°), so during operation it should point towards the location of IR sensor on the fan.


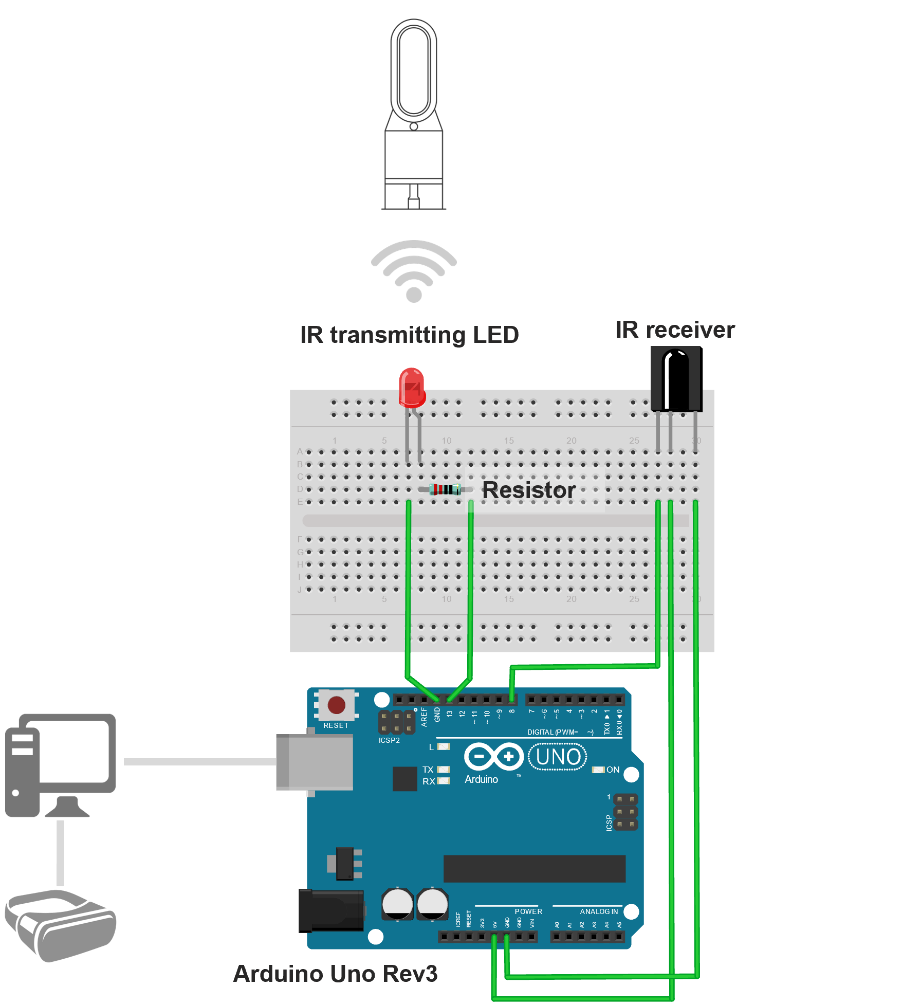


**Figure 3. Schematic Diagram for Connections in Wind Control Module. Note: Components in this figure are not on the same scale. Elements are resized to show more details.**

**
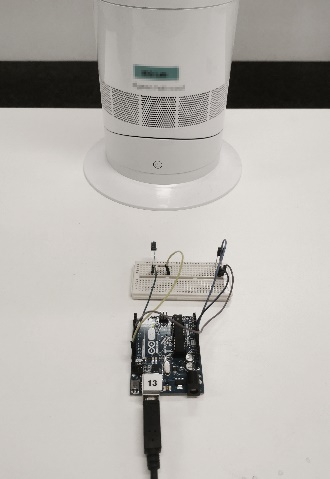
**

**Figure 4. Wind Control Module Prototype**

***Step two: Programming Arduino***

The Arduino Uno Rev3 was programmed in the Arduino IDE (based on C++) with two scripts, *IR signal receiver* and *IR signal sender* (see supplement files). *IR signal receiver* was written to receive signal from the IR LED receiver (pin 8 on Arduino Uno Rev3) while the IR signal sender to send signal via the Infrared Transmitting LED to the fans.

1. Connect Arduino Uno Rev3 to PC via USB.
2. Put *Types.h* (c++ header file where the variables are declared),*IR signal receiver* and *IR signal sender* files into the same folder.
3. Open and upload *IR signal receiver* script to Arduino control board using Arduino IDE.
4. Open *Serial Monitor* in *Tools.*
5. Send command “*r*” in the *Serial Monitor* window to activate the IR signal receiver function.
6. Point the fan’s remote control to the IR signal receiver and click ON/OFF button on the remote. A serial number will appear which is the encoded signal can be used by the infrared LED to send specific commands to the fan.
7. Repeat step 4-5 to get every serial number of all the functions on the remote. In this case, the remote has function of turning up and down the fan speed.
8. Upload *IR signal sender* script to Arduino UNO Rev3.
9. Test if the serial number works. (add “*s*” at the beginning and “*0*” at the end of all the serial numbers for sending the encoded infrared signal. “*s*” activates the *IR signal sender* function in the script and “*0*” indicates end of command.

***Step three: Calibrate the fan and generate airflow commands***

1. Determine fan’s location in relation to the user.

The relative position of the fan in relation to the VR user should be calibrated with an anemometer, matching the air velocity measured at the fan’s highest and lowest operating levels with the maximum and minimum target airflow velocity (m/s). Note: *Use the maximum air velocity to determine the distance of the fan to the user. If the minimum air velocity cannot be matched with the air velocity at the fan’s lowest operating level, more fans are needed.*

1. Generate airflow profile commands

The target airflow velocity should be converted to discrete air velocity levels, responding to different fan operating levels. The previous signal commands for increase and decrease fan speed were given variable names in the C# script in Unity: *Inc ()* and *Dec ()*. The discreate air velocity level sequence can be converted to a series of commands with *Inc ()* and *Dec ().* For example, if air velocity level change from level 1 to 4 in a second, three *Dec ()* commands will be used. Note: variable *Inc ()* and *Dec ()* will be defined in the C# script in step four.

***Step four: Integration into Unity***

C# script can be executed during Unity VR runtime. A function *Wind Script* (see supplement files) in C# was created to be able to send commands to the programed Arduino UNO Rev3 directly from *Unity* (instead of from Arduino IDE). In this way, the commands for controlling the fan to change operating levels in order to simulate target airflow can be embedded in Unity’s platform and this function can be called from other actions in VR. For example, *Inc()* and *Dec()* were defined to speed up and down the fan’s operating levels. Note: In this case, if the user enters a *wind* zone in the VR environment, defined as the collision between the GameObject of the player and the GameObject of the *wind* zone, an event will be triggered to active the *wind profile* function in *wind script*. If the user exits that zone, *CloseFan* function will be executed. *OnTriggerEnter* and *OnTriggerExit* (Unity technology, 2022) were used to achieve this*.* Step-by-step procedures are listed below.

1. Create an empty GameObject *wind* in *Unity* and attach the script *Wind Script* to the GameObject. Fill in the empty fields under the *Wind Script* tab, including Serial Port Name and serial numbers for all the fan operation commands.
2. In *Wind Script*, copy the airflow profile commands from Step three (2) to the *wind profile* function. Note: If there are multiple airflow profiles, multiple *wind profile* functions should be created with the respective commands belonging to different airflow profiles*.*
3. Create microclimatic zones for different wind profiles. Depending on the shape of the microclimate zones, box collider or mesh collider can be used. Attach the *wind zone* script (see supplement files) onto this object. Relevant wind profiles can be activated in this script. Note: If the shape is irregular, a 3D geometric mesh model for that zone needs to be modelled and imported into Unity to create a GameObject. Mesh collider function can be attached to that GameObject.
4. Attach a *Capsule Collider* (its shape is two half-spheres joined together by a cylinder to represent virtual human body) and *Rigidbody* component to the VR user’s avatar GameObject (using VR, this would be the *XR Rig* GameObject, see https://docs.unity3d.com/Manual/configuring-project-for-xr.html). Enable *Collider.isTrigger* and *Rigidbody.isKinematic* (Unity technology, 2022).

**3.2 Virtual sun**

Simulation of the virtual sun in VR is achieved by using a bank of 10 quartz tungsten halogen lamps (250W ×10) mounted on a 0.6m × 0.9m frame. Radiative heat from the halogen lamp offers good approximation to that of the solar radiation because of its close match to the infrared waveband in the solar spectrum (Tawfik et al., 2018; Yu, 2020). The operation of halogen lamps (ON/OFF) is controlled by the *Sunlight Control Module* for user interaction in the VR environment. Comprising of two sets of Arduino microcontrollers (Arduino UNO Rev3), prototyping board shields and power relays (DuinoTECH XC-4419), the *Sunlight Control Module* can receive signals from the triggered events in *Unity* and control the status of the heat lamps to simulate a dynamic experience of sunlight.

***Hardware and Software Components:***

**Table 2. List of Items Required for Sunlight Simulation in VR**

| **Hardware** | | | |
| --- | --- | --- | --- |
| Item | Model | | Quantity |
| Virtual Reality headset | Oculus Rift S | | 1 |
| VR compatible computer | Lenovo | | 1 |
| Halogen Lamps |  | | 10 |
| Arduino microcontroller | Arduino Uno Rev3 | | 2 |
| Arduino prototyping board shields | XC4482 | | 2 |
| Power relay | DuinoTECH XC-4419 | | 2 |
| Power socket | Tesla 10 Amp Double Pole Double Internal Powerpoint White | | 2 |
| Electrical Cable | Olex 2.5mm Two Core and Earth Cable | | 1m |
| Power plug |  | | 1 |
| Protection box | Sealed Polycarbonate Enclosure (171 x 121 x 55 mm) | | 1 |
| Soldering station |  | | 1 |
| Electrical System | Alternating Current system (220-240 Volts, 50Hz) | |  |
| **Software** | | | |
| Unity 2022 |  |  | |
| Visual Studio 2019 |  |  | |
| Arduino IDE 2.0 |  |  | |

***Step one: Hardware set-up***

The *Sunlight Control Module* works as a digitally controlled power relay for the operation of the halogen lamps. Instead of using the Solderless Breadboard, prototyping shields were used to provide a permanent configuration of the circuit. In addition, the control module was mounted into a sealed polycarbonate box which has openings for connections to computer and power source. Therefore, the need for physical access to the circuit can be minimized for electrical safety (as direct current is controlled via this module). No resistor is added in the circuit because the power relay contains one. The capacity of the power relay (10Amp 250 Volts capable of supporting 2500 watts external device) to control the halogen lamps was checked.

***
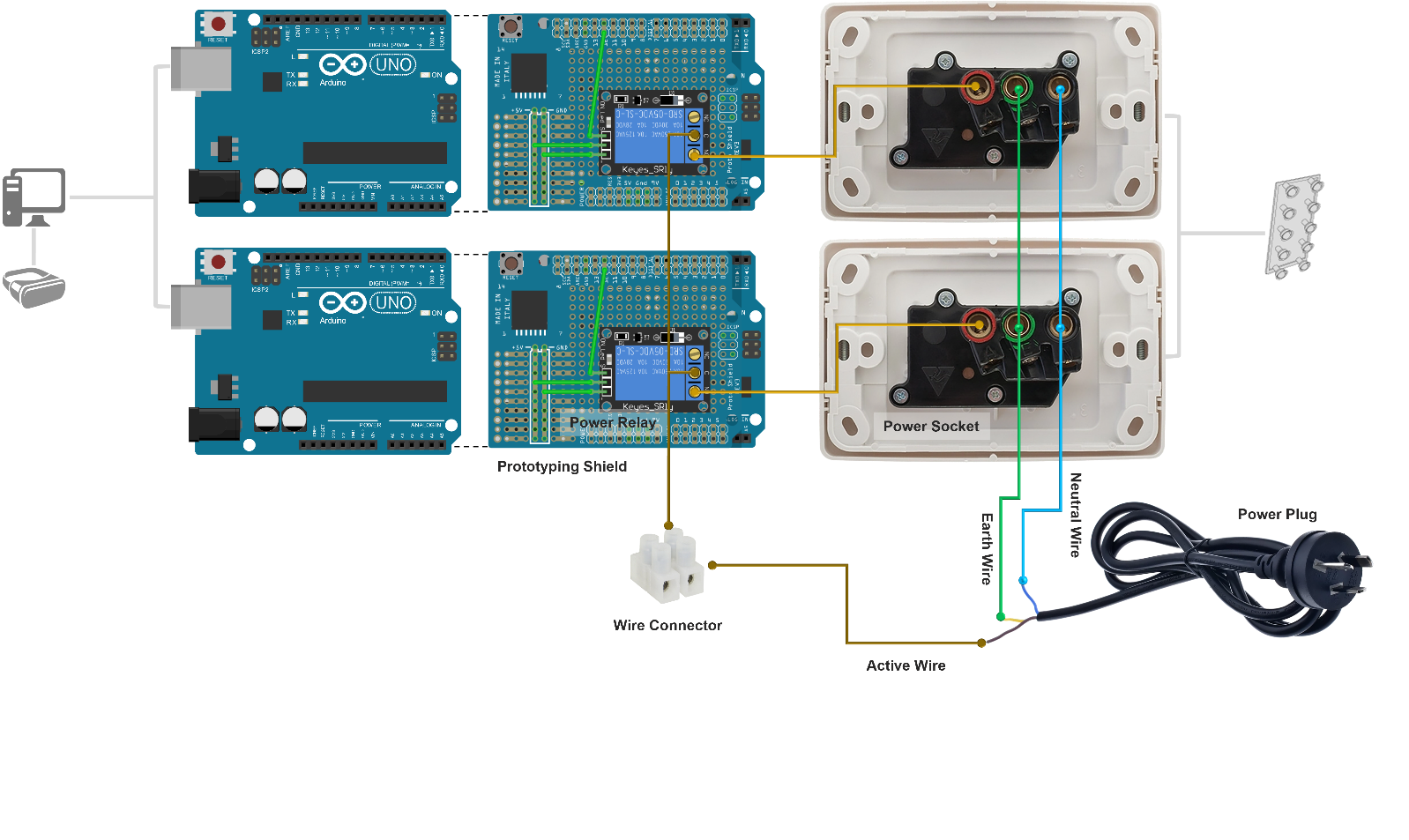
***

**Figure 5. Schematic Diagram for Connections in Sunlight Control Module. Note: Components in this figure are not on the same scale. Elements are resized to show more details.**

***
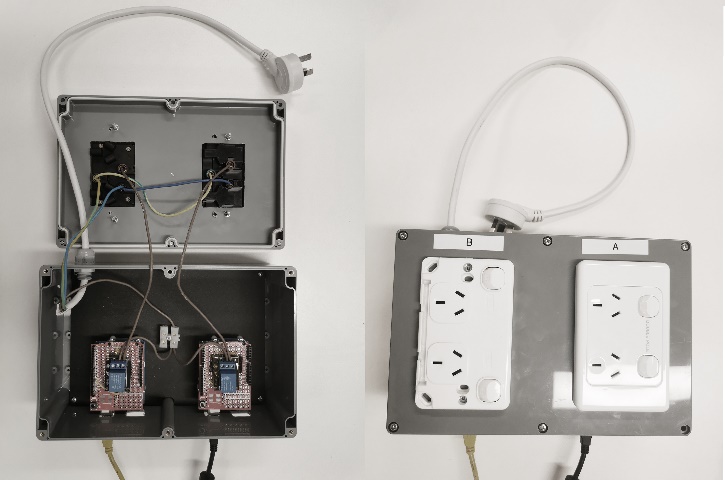
***

**Figure 6. Sunlight Control Module Prototype**

1. Connect the power relay to the Arduino prototyping board shield with soldering. Note: Connect *S* to any of the digital pin in the prototyping shield (in the example, pin 12 was used), “+” to the *5V* pin, and “-” to the *Ground* pin.
2. Create openings in the Sealed Polycarbonate Enclosure for the power socket boards, power plug and USB connection. Fix the power socket boards on to the box.
3. Connect the power relay, power socket boards and power plug with electrical cable. Note: Connect the “*C*” (Common) end of power relay to the *Active* wire of the power plug, “*NO*” (Normally Open) end of the power relay to the *Active Terminal* of the power socket, the *Earth* wire of the power plug to the Earth Terminal of the power socket and the Neutral wire of the power plug to the Neutral Terminal of the power socket.
4. The Arduino prototyping board shield needs to be plugged onto the Arduino Uno Rev3.
5. The Arduino Uno Rev3 with the prototyping shield plugged should be mounted onto the base of the protection box.
6. Connect *Sunlight Control Module* to the PC via USB, to the halogen lamps and to Direct Current supply.
7. The location of the halogen lamps in relation to the VR user needs to be determined based on the specific requirements. Radiation flux density may be measured for this purpose.

***Step two: Programming Arduino***

Programming for the *Sunlight Control Module* consists of the functionality for receiving serial commands from the USB port in PC with the *Serial.readBytesUntil* (Arduino, 2022c) function and activate specific halogen lamp operation definitions. Note: Halogen lamp operation mode is defined using *digitalWrite* (Arduino, 2022b) which writes a HIGH or a LOW value to a digital pin connecting the power relay (HIGH will cause power relay to open the circuit whereas LOW will cause it to close the circuit). For example, when receiving a character value of “O”, digitalWrite will output *HIGH* (*1* in digital signal) value to open the circuit for five halogen lamps. The script *Heatlamp-arduino-script* (see supplement files) needs to be uploaded to Arduino via Arduino IDE.

***Step three: Integration into Unity***

The hardware configuration can simulate three sunlight conditions: *shaded* (all halogen lamps off), *partially* *shaded* (five halogen lamps off) and *sunlit* (all halogen lamps on). These are defined as three functions in the script to control the operation of the halogen lamps by sending specific serial command to the *Sunlight Control Modules*, e.g., sending serial commands “*C*” to turn off power connection to two sets of halogen heat lamps. These functions can be called by event trigger in Unity, either by Unity’s User Interface or by user enter/exit certain areas.

1. Create an empty GameObject named *Sunlight*. Attach the script *Artificial Sun Script* onto this GameObject, fill in port names for the USB port used, e.g., COM1. Note: the port names can be found in *Device Manager* function in Windows operating system.
2. Create microclimatic zones for different sunlight conditions. Depending on the shape of the microclimate zones, box collider or mesh collider can be used. Attach the *TriggerSunlit, TriggerShade* and *TriggerPartialShade* scripts (see supplement files) to the respective microclimate GameObjects. Note: If the shape is irregular, a 3D geometric mesh model for that zone needs to be modelled and imported into Unity to create a GameObject. Mesh collider function can be attached to that GameObject. Drag and place the *Sunlight* GameObject into the field *Artificial Sun*.
3. Attach a *Capsule Collider* (its shape is two half-spheres joined together by a cylinder to represent virtual human body) and *Rigidbody* component to the VR user’s avatar GameObject (using VR, this would be the *XR Rig* GameObject, see https://docs.unity3d.com/Manual/configuring-project-for-xr.html). Enable *Collider.isTrigger* and *Rigidbody.isKinematic* (Unity technology, 2022).

# Limitation and Recommendation

This research develops a method of integrating immersive thermal experience into Virtual Reality with airflow and radiation simulation. The multisensory VR system is capable of simulating the outdoor dynamic thermal experience and affording user interaction with the thermal environment via the Arduino-based control modules. Limitations that demand future development are related to 1) the Arduino control module and 2) the sensory output devices: The Arduino control module for the virtual sun uses a power relay to control the ON/OFF status of the halogen lamps which is only a binary operation. Control of voltage for the power supply to achieve a continuous change of radiation from the halogen lamp can be future investigated, potentially with pulse-width modulation (PWM) method. Furthermore, only halogen lamps and bladeless fans were used in the prototypes. More sensory output devices, such as mist fans for humidity delivery, conductive heating or cooling devices and scent delivery devices, can be tested with the same principle described in this article.

**Ethics statements**

The relevant informed consent was obtained from subjects participated in this study.

**CRediT author statement**

**Kun Lyu:** Conceptualization, Methodology, Software, Writing - Original Draft.  **Richard de Dear**: Supervision, Writing - Review & Editing. **Anastasia Globa:** Supervision, Writing - Review & Editing, Software. **Arianna Brambilla**: Supervision, Writing - Review & Editing.

**Acknowledgments**

This research did not receive any specific grant from funding agencies in the public, commercial, or not-for-profit sectors.

**Declaration of interests**

Please **tick** the appropriate statement below (please do not delete either statement) and declare any financial interests/personal relationships which may affect your work in the box below.

The authors declare that they have no known competing financial interests or personal relationships that could have appeared to influence the work reported in this paper.

The authors declare the following financial interests/personal relationships which may be considered as potential competing interests:

*Please declare any financial interests/personal relationships which may be considered as potential competing interests here.*

**Supplementary material *and/or* additional information [OPTIONAL]**

Scripts for Arduino:

- IR signal receiver.ino
- IR signal sender.ino
- types.h
- Heatlamp-arduino-script.ino

Scripts for Unity – Arduino Interaction:

- WindScript.cs
- WindZone.cs
- ArtificalSunScript.cs
- TriggerShade.cs
- TriggerSunlit.cs
- TriggerPatialShade.cs

# Reference

Alamirah, H., Schweiker, M., & Azar, E. (2022). Immersive virtual environments for occupant comfort and adaptive behavior research – A comprehensive review of tools and applications. *Building and Environment*, *207*, 108396. https://doi.org/10.1016/j.buildenv.2021.108396

Arduino. (2022a). *Arduino—Home*. https://www.arduino.cc/

Arduino. (2022b). *digitalWrite()—Arduino Reference*. https://www.arduino.cc/reference/en/language/functions/digital-io/digitalwrite/

Arduino. (2022c). *Serial.readBytesUntil()—Arduino Reference*. https://www.arduino.cc/reference/en/language/functions/communication/serial/readbytesuntil/

Brengman, M., Willems, K., & De Gauquier, L. (2022). Customer Engagement in Multi-Sensory Virtual Reality Advertising: The Effect of Sound and Scent Congruence. *Frontiers in Psychology*, *13*. https://www.frontiersin.org/article/10.3389/fpsyg.2022.747456

Cooper, N., Milella, F., Pinto, C., Cant, I., White, M., & Meyer, G. (2018). The effects of substitute multisensory feedback on task performance and the sense of presence in a virtual reality environment. *PLOS ONE*, *13*(2), e0191846. https://doi.org/10.1371/journal.pone.0191846

Ergan, S., Radwan, A., Zou, Z., Tseng, H., & Han, X. (2019). Quantifying Human Experience in Architectural Spaces with Integrated Virtual Reality and Body Sensor Networks. *Journal of Computing in Civil Engineering*, *33*(2), 04018062. https://doi.org/10.1061/(ASCE)CP.1943-5487.0000812

Kim, D., Bang, J., Lee, W., Ha, I., Lee, J., Eom, H., Kim, M., Park, J., Choi, J., Kwon, J., Han, S., Park, H., Lee, D., & Ko, S. H. (2020). Highly stretchable and oxidation-resistive Cu nanowire heater for replication of the feeling of heat in a virtual world. *Journal of Materials Chemistry A*, *8*(17), 8281–8291. https://doi.org/10.1039/D0TA00380H

Kuliga, S. F., Thrash, T., Dalton, R. C., & Hölscher, C. (2015). Virtual reality as an empirical research tool—Exploring user experience in a real building and a corresponding virtual model. *Computers, Environment and Urban Systems*, *54*, 363–375. https://doi.org/10.1016/j.compenvurbsys.2015.09.006

Lee, J., Kim, D., Sul, H., & Ko, S. H. (2021). Thermo-Haptic Materials and Devices for Wearable Virtual and Augmented Reality. *Advanced Functional Materials*, *31*(39), 2007376. https://doi.org/10.1002/adfm.202007376

Lee, J., Sul, H., Lee, W., Pyun, K. R., Ha, I., Kim, D., Park, H., Eom, H., Yoon, Y., Jung, J., Lee, D., & Ko, S. H. (2020). Stretchable Skin-Like Cooling/Heating Device for Reconstruction of Artificial Thermal Sensation in Virtual Reality. *Advanced Functional Materials*, *30*(29), 1909171. https://doi.org/10.1002/adfm.201909171

Lyu, K., Brambilla, A., Globa, A., & de Dear, R. (2023). An immersive multisensory virtual reality approach to the study of human-built environment interactions. *Automation in Construction*, *150*, 104836. https://doi.org/10.1016/j.autcon.2023.104836

Oh, J., Kim, S., Lee, S., Jeong, S., Ko, S. H., & Bae, J. (2021). A Liquid Metal Based Multimodal Sensor and Haptic Feedback Device for Thermal and Tactile Sensation Generation in Virtual Reality. *Advanced Functional Materials*, *31*(39), 2007772. https://doi.org/10.1002/adfm.202007772

Pyun, K. R., Rogers, J. A., & Ko, S. H. (2022). Materials and devices for immersive virtual reality. *Nature Reviews Materials*, *7*(11), Article 11. https://doi.org/10.1038/s41578-022-00501-5

Sanchez-Vives, M. V., & Slater, M. (2005). From presence to consciousness through virtual reality. *Nature Reviews Neuroscience*, *6*(4), 332–339. https://doi.org/10.1038/nrn1651

Smith, J. W. (2015). Immersive Virtual Environment Technology to Supplement Environmental Perception, Preference and Behavior Research: A Review with Applications. *International Journal of Environmental Research and Public Health*, *12*(9), Article 9. https://doi.org/10.3390/ijerph120911486

Tawfik, M., Tonnellier, X., & Sansom, C. (2018). Light source selection for a solar simulator for thermal applications: A review. *Renewable and Sustainable Energy Reviews*, *90*, 802–813. https://doi.org/10.1016/j.rser.2018.03.059

Tseng, K. C., & Giau, D. T. N. (2022). A feasibility study of using virtual reality as a pre-occupancy evaluation tool for the elderly. *Automation in Construction*, *134*, 104037. https://doi.org/10.1016/j.autcon.2021.104037

Yu, Y. (2020). *Thermal respite for pedestrians in overheated urban environments* [Thesis, University of Sydney]. https://ses.library.usyd.edu.au/handle/2123/24302

1. The use of two fans in this case study is necessary to achieve the required range of air velocity for simulating the wind sequence. For airflow with less turbulence, a single fan may be sufficient. [↑](#footnote-ref-2)
